# Data driven approach to identify vulnerable plaques in carotid atherosclerosis using computed tomography imaging and computational fluid dynamics: A pilot study

**DOI:** 10.1371/journal.pdig.0001762

**Published:** 2026-10-07

**Authors:** Shaokai Zheng, David Bervini, Hugo Besson, Sofía González Martínez, Mirjam R. Heldner, Mandy Müller, Thomas Meinel, Johannes Kaesmacher, Andreas Harloff, Dominik Obrist

**Affiliations:** 1 ARTORG Center for Biomedical Engineering Research, University of Bern, Bern, Switzerland; 2 Department of Diagnostic and Interventional Neuroradiology, Inselspital, Bern University Hospital and University of Bern, Bern, Switzerland; 3 Department of Neurology, Inselspital, Bern University Hospital and University of Bern, Bern, Switzerland; 4 Department of Neurosurgery, Inselspital, Bern University Hospital and University of Bern, Bern, Switzerland; 5 Department of Neurology and Neurophysiology, Faculty of Medicine, Medical Center-University of Freiburg, Freiburg, Germany; Massachusetts Institute of Technology, UNITED STATES OF AMERICA

## Abstract

The current clinical gold standard for identifying vulnerable plaque is plaque Magnetic Resonance Imaging (MRI). However, owing to its limited availability, many patients do not have access. This pilot study aims to explore whether computational fluid dynamics (CFD) modelling, established using computed tomography angiography (CTA) and neurovascular ultrasound measurements (NVUS), as part of current clinical routine, can be used to triage vulnerable plaque in patients with carotid atherosclerotic stenosis. Nine patients who underwent evaluation for carotid endarterectomy between January 2021 and March 2023 were retrospectively included. Carotid geometries were segmented from CTA and reconstructed for CFD simulations using patient-specific NVUS-derived boundary conditions. Geometric and novel hemodynamic features were extracted. A logistic regression (LR) and a support vector machine (SVM) classifier with leave-one-out cross-validation and embedded feature selection were trained to identify vulnerable plaque, assessed according to the Plaque-Reporting and Data System based on multi-modal plaque MRI findings. Model performance was evaluated using area under the receiver operating characteristic curve (ROC-AUC), precision-recall AUC, confusion matrix, and other related metrics. Eighteen carotid arteries (9/18 were vulnerable) were analyzed. The classifiers achieved mean ROC-AUC of 0.77 and 0.78 for the LR and SVM, respectively. Key risk factors identified by both classifiers included mean helicity (OR: 0.5, 95%CI: 0.42, 0.58; mean feature coefficient: -0.27, 95%CI: -0.39, -0.15), kurtosis/skewness of helicity, and skewness of streamwise vorticity. Patient-level and sub-cohort analyses also support these findings. This pilot study demonstrated that combining NVUS and CTA with CFD may facilitate the identification of vulnerable plaques. Higher order statistics of intravascular hemodynamics may serve as promising surrogate features of plaque vulnerability.

## Author summary

This study explored a new way to identify vulnerable carotid plaque, which contributes to one in every five stroke cases. Currently, the best method for detecting these plaques is through plaque magnetic resonance imaging (MRI), but it is unavailable in many clinics. In this study, we used a combination of standard clinical imaging modalities and computer modeling methods to simulate blood flow in the arteries. Information about the blood flow was extracted from these results, and machine learning models were trained to predict which patients had vulnerable plaques. Our results indicate that our method has the potential to identify high-risk cases for patients without access to MRI and might help reduce the risk of stroke. This approach could eventually offer a more accessible alternative to MRI for identifying dangerous carotid plaques.

## 1. Introduction

Carotid atherosclerotic stenosis (CAS) affects approximately 3–6% of people over 70 years old and worsens with age [1]. It accounts for approximately one in five patients with ischemic stroke or transient ischemic attack [2]. The primary mechanism underlying cerebral ischemia caused by CAS is the rupture of an ipsilateral plaque followed by a thrombo-embolic event. This has fostered the concept of vulnerable or high-risk plaque, which is prone to embolism [3–6]. Since vulnerable plaques have a prevalence of about 26% in patients with asymptomatic CAS [6], identifying such plaques is crucial for selecting patients for endarterectomy to prevent thrombo-embolic events.

Previously, in the North American Symptomatic Carotid Endarterectomy Trial (NASCET), patients were selected based on the degree of stenosis, ranging from 30% to 99% [7]. With the advancement of imaging tools, differentiation of plaque composition has further facilitated the identification of vulnerable plaque [8]. High-risk features such as fibrous cap ulceration, lipid-rich necrotic core, and intra-plaque hemorrhage have emerged as strong predictors of thrombo-embolic events, independent of the degree of stenosis [9,10]. As a result, the focus of risk stratification has shifted from quantifying stenosis [11,12] to characterizing plaque [9,13]. Identifying high-risk patients has become increasingly important, given the overall neutral effect of revascularization observed in the Second European Carotid Surgery Trial (ECST-2) [14] – at least for patients with a low to intermediate predicted risk of stroke.

In this regard, extensive comparisons have been made regarding the diagnostic power of each imaging modality [8,15]. Color-coded duplex sonography alone has shown very limited efficacy in predicting plaque progression [16], but it remains the first-line imaging modality in clinical routine due to its high availability. Computed tomographic angiography (CTA) has demonstrated higher sensitivity than magnetic resonance angiography (MRA), along with a higher positive predictive value for detecting stenosis and occlusion [17]. In contrast, MR-detected intra-plaque hemorrhage has been shown to be an independent and strong predictor of thrombo-embolic events [18,19]. Such features may be inferred from the soft plaque thickness and the ‘rim sign’ on CTA images [20], although clinical evidence remains debated [21]. Hence, plaque MR imaging continues to be the preferred modality for plaque risk assessment [13,22].

In recent years, the clinical adoption of 4D flow MR (4DMR) sequences has enabled in vivo studies of the hemodynamic characteristics associated with CAS plaques. Wall shear stress (WSS) along with its derived forms has been shown to correlate strongly with plaque vulnerability and progression [23–25]. Indeed, WSS influences the behavior of endothelial cells and, indirectly, the synthetic and contractile activity of underlying smooth muscle cells, playing a major role in the pathophysiological processes of the vascular wall in steno-occlusive disease [26–29]. However, due to the limited availability of MR scanners and 4DMR sequences, clinical evidence remains scarce.

To bridge the gap, computational fluid dynamics (CFD) methods have been leveraged to simulate blood flow within the carotid arteries (CA) and extract hemodynamic characteristics [30–33]. Using the geometries of the CA and corresponding boundary conditions (BCs) at the common (CCA), internal (ICA), and external carotid arteries (ECA), the luminal velocity field can be solved with high accuracy, allowing for the derivation of detailed hemodynamic parameters. Most studies have focused on WSS, and, although findings are controversial [34], lower and more oscillatory WSS have been correlated with plaque formation [23] and destabilization [35]. However, to clinically validate these associations, prospective cohort studies are needed. As a result, evidence supporting the use of CFD to identify patients with high-risk carotid plaques remains preliminary.

In this study, we designed a pilot cross-sectional analysis to explore whether CFD modelling, established using CTA and Doppler ultrasound measurements, as part of current clinical routine, can be used to triage vulnerable plaque in patients with CAS, in comparison with gold-standard plaque MR imaging evaluation. We extracted intra-vascular hemodynamic features and explored their descriptive power with two machine learning classifiers. Upon further validation with multicentric data, the proposed workflow could be incorporated into a recommendation system and ultimately benefit patients in resource-limited settings.

## 2. Materials and methods

### 2.1 Ethics statement

The original cohort was collected as part of a study to investigate candidate biomarkers of atherosclerotic plaque vulnerability in endarterectomy specimens of patients with carotid disease (ethical approval by the local ethics committee, KEK Bern, No.2020-03019). Inclusion and exclusion criteria and imaging protocols are detailed in the S1 Appendix (Tables A and B). The patient cohort in this study was derived from the original cohort with all patient-related information anonymized, and ethical approval was obtained from the local ethics committee (KEK Bern, No.2023-01278), covering retrospective use of the anonymized data. All procedures performed in this study were in accordance with the ethical standards of the institutional committees and with the Helsinki Declaration.

### 2.2 Patients

The current cohort was derived from the original cohort by screening consecutive patients scheduled for carotid endarterectomy at Inselspital, Bern University Hospital with either symptomatic or asymptomatic CAS between January 2021 and March 2023. Inclusion criteria were based on the availability of pre-operative neurovascular ultrasound (NVUS), neck CTA, and plaque vulnerability rating based on MRI. Patients were excluded if any of the pre-requisites were missing, or if NVUS reading was incomplete to set up the CFD. Reporting of this study follows the Checklist for Artificial Intelligence in Medical Imaging (CLAIM) [36] and the STARD 2015 guideline [37].

### 2.3 Clinical data

The current standard of care mandates NVUS measurements of blood flow velocities in the CCA, ICA, and ECA. At our institution, NVUS examinations were performed using color-coded duplex sonography with a standard ultrasound system (Canon Aplio 500, Canon Medical, Japan) equipped with a linear array transducer (9 MHz). The arteries were assessed in B-mode as well as with color and pulsed Doppler imaging. Peak systolic and diastolic velocities were measured, and vessel wall morphology was evaluated. For peak systolic velocity measurements, the probe was moved along the vessel – from proximal to distal of the stenosis to register the highest velocity. The average diastolic velocity was also recorded. All NVUS examinations were conducted by experienced neurovascular sonographers certified by the Swiss Society of Clinical Neurophysiology.

All CTA and MRI images were acquired at the Department of Diagnostic and Interventional Neuroradiology, Inselspital, University Hospital Bern, following the clinical protocol of routine imaging work up of CAS patients. No further quality control was implemented to preserve the possible variance intrinsic to the real-world data. For this study, we used exclusively CTA images, acquired on multiple scanners (SIEMENS SOMATOM, Siemens Healthineers, Germany; GE Optima, GE Healthcare, US; Canon Aquilion, Canon Medical, Japan; Philips Brilliance, Philips Healthcare, NL) with a tube voltage of 100 kVp. The raw DICOM images were converted to NIFTI format and segmented semi-automatically using the active contour method implemented in the ITK-SNAP (v4.0.2). To do that, CTA images were thresholded between 150 and 1000 Hounsfield units. Then a total of four seeds were placed at the bifurcation and in each of the CCA, ICA, and ECA branches. The same process was repeated for each side of the patient. After that, approximately 400 iterations were executed with the active contour algorithm. Labels with other branches, such as the superior thyroid, lingual and facial arteries, were removed post-hoc. The resulting binary mask was used to extract the geometry, and the marching cubes algorithm from scikit-image (v0.19.3) was applied to reconstruct the surface with the Lewiner method [38] and a step size of two. Subsequently, Taubin smoothing (pyvista, v0.40.4) was employed to condition the surface with default settings for ten iterations, and the final carotid geometry surface was exported. Examples of the segmented CTA images and reconstructed geometries are shown in Fig 1.

**Fig 1:**
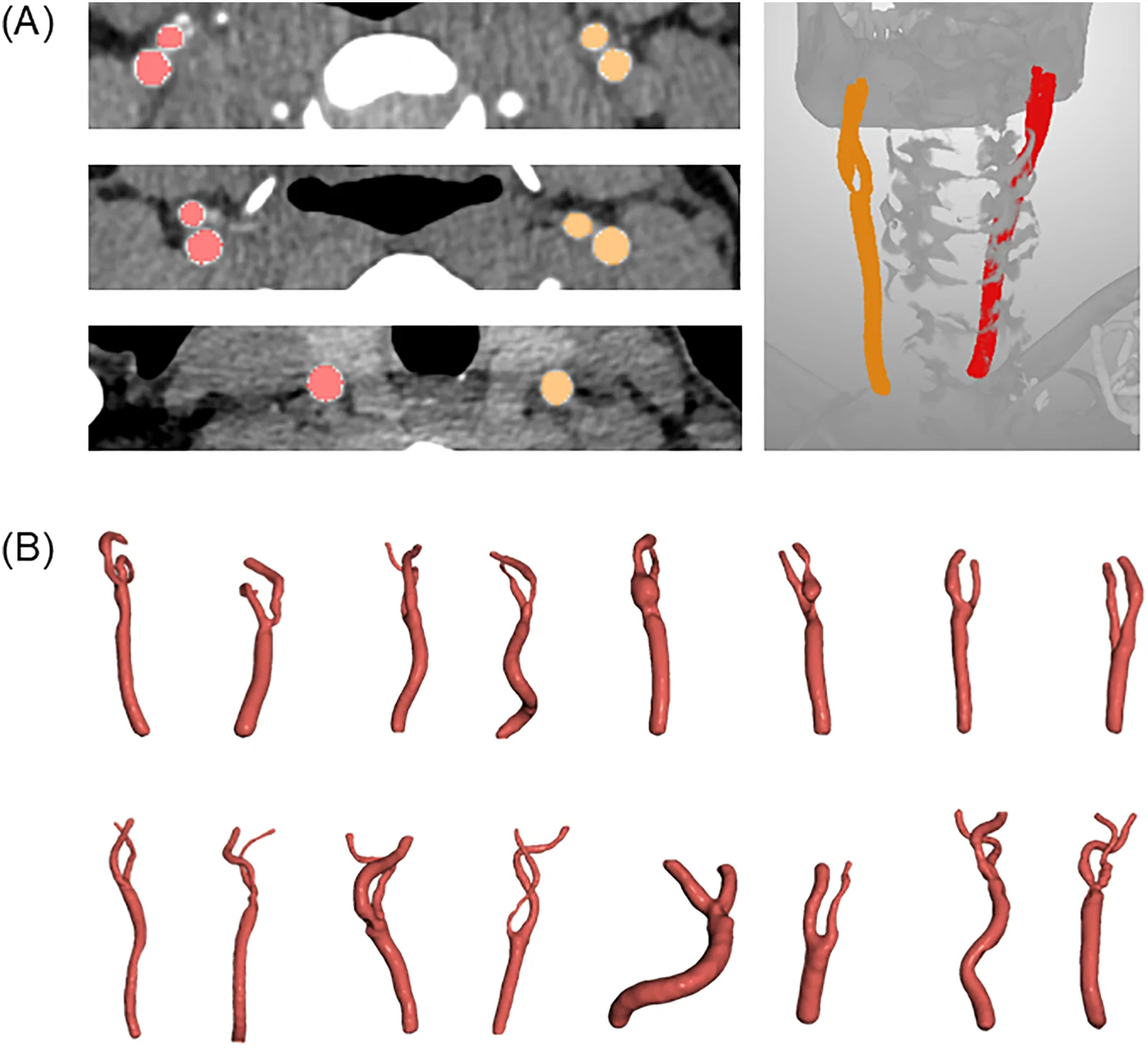
(A) Raw CTA images overlaid with segmentation masks (yellow: left; red: right) in slices and 3D view. (B) Reconstructed carotid geometries (not to scale).

The binary classifications of the carotid were extracted from the structured clinical neuroradiology report, embedded in an internal structured operating procedure of the diagnostic neuroradiology team. Patients scheduled for carotid endarterectomy underwent a multi-modal MRI of the carotid artery and the brain as part of clinical routine prior to the procedure. The MRI routine typically consists of a 3D- time-of-flight (TOF), and a pre- and post-contrast T1 turbo spin echo (TSE) sequence of cervical arteries. Furthermore, diffusion weighted imaging (DWI) of the brain to detect acute ischemic brain lesions and fluid-attenuated inversion recovery (FLAIR) sequences to assess age-related white matter changes and chronic ischemic brain lesions are routinely performed. As part of the study, consenting patients additionally underwent a three-dimensional magnetization-prepared rapid acquisition gradient recalled echo sequence (MPRAGE) with fat suppression to identify intra-plaque hemorrhage (IPH). This study-specific MRI sequence was performed as part of the routine MRI scan, adding approximately four minutes to the scan time. MRI scans were analyzed centrally. In essence, radiological features including intra-plaque hemorrhage, ruptured fibrous cap, and intraluminal thrombus were rated as vulnerable plaque aligned with the grade 4a-4c of the Plaque-RADS grading system as indications of high risk of ipsilateral cerebrovascular events [39]. A summary of the imaging findings in the grading system is included in Table 1, while definitions of the imaging biomarkers can be found in the reference [39].

**Table 1:** Summary of the Plaque-RADS grading system, reproduced after Saba et al. [39]. MWT: maximum wall thickness; LRNC: lipid-rich necrotic core; FC: fibrous cap; IPH: intra-plaque hemorrhage; RADS: Reporting and Data System.

Plaque-RADS Score	Attributable Risk of Ipsilateral Cerebrovascular Events	Imaging Findings
**1**	Absent	Normal vessel wall
**2**	Low	MWT < 3 mm
**3**	Moderate	MWT ≥ 3 mm or healed ulcerated plaque
**3a**	Moderate	LRNC with intact thick FC (MWT ≥ 3 mm)
**3b**	Moderate	LRNC with thin FC (MWT ≥ 3 mm)
**3c**	Moderate	Healed ulcerated plaque
**4**	High	Complicated plaque (irrespective of MWT)
**4a**	High	IPH
**4b**	High	Ruptured FC
**4c**	High	Intraluminal thrombus

### 2.4 Computational fluid dynamics

To prepare the geometry of the carotid bifurcation for CFD simulation, each branch was truncated using cut planes at the inlet (CCA) and outlets (ICA, ECA), perpendicular to their respective local centerlines. The domain was then discretized with tetrahedral elements, including three boundary layer elements with a stretching factor of 1.2, resulting in 1.3 to 1.8 million cells per carotid geometry.

To set the BCs, the flow rates of the CCA and ICA branches were prescribed using the equation $Q=0.5U*A=\text{0}.\text{125}\pi UD^{\text{2}}$, where U is the velocity measured from NVUS, A is the area, and D is the diameter of the corresponding local branch. The diameter of the CCA was measured at three different longitudinal locations, and the average was used to calculate the flow rate ($Q_{CCA}$). For the flow rate in ICA ($Q_{ICA}$), the minimum diameter was used, assuming that the maximum velocity measured by NVUS occurred at the narrowest part of the artery. For each subject, peak systolic and diastolic flow rates in the CCA and ICA were defined based on the NVUS measurements. The outlet of the ECA was prescribed as a stress-free boundary condition with backflow suppression as implemented in COMSOL Multiphysics (V6.1), where the outlet pressure is adjusted to prevent fluid from entering the domain through the boundary.

After setup, the flow field was solved using the finite element software COMSOL. The Navier-Stokes equations were solved using the laminar flow model, which is appropriate for the flow conditions typically observed in carotid arteries (typically$Re_{D}\ll \text{2}\text{3}\text{0}\text{0}$) and considerations for transitional flow or turbulence effects may be omitted [30,31,40–42]. Steady-state solutions were computed using the peak systolic and diastolic flow rates, respectively. No pulsatile BC was prescribed because it exceeds the current clinical protocol and simulated idealized pulsatile profile may lead to false representations of hemodynamic features [43]. Blood was modeled as a Newtonian fluid with a density of $\rho =\text{1}\text{0}\text{6}\text{0}kg/m^{\text{3}}$ and a viscosity of $\mu =\text{3}.\text{7}\text{1}\times {\text{10}}^{-\text{3}}\text{ Pa}\cdot \text{s}$, parameters shown to yield reasonable results in carotid blood flow simulations [33]. Although non-Newtonian blood models have been used in previous studies [33], a lack of evidence in choosing the appropriate model, e.g., Carreau-Yasuda, Casson, and Power-Law, hinders their rational adoption without evaluating the associated uncertainties on patient-specific data. Further, it has been reported that for large and medium sized vessels, such as CA, a Newtonian assumption is considered reasonable [33]. The inlet velocity profile was set to parabolic. As discussed in a previous study by comparing uniform, Womersley, and parabolic velocity profiles [41], the parabolic velocity profile provides results most similar to 4DMR-derived patient-specific flow. The schematic workflow is illustrated in Fig 2A, with suggested clinical workflow (Fig 2B).

**Fig 2:**
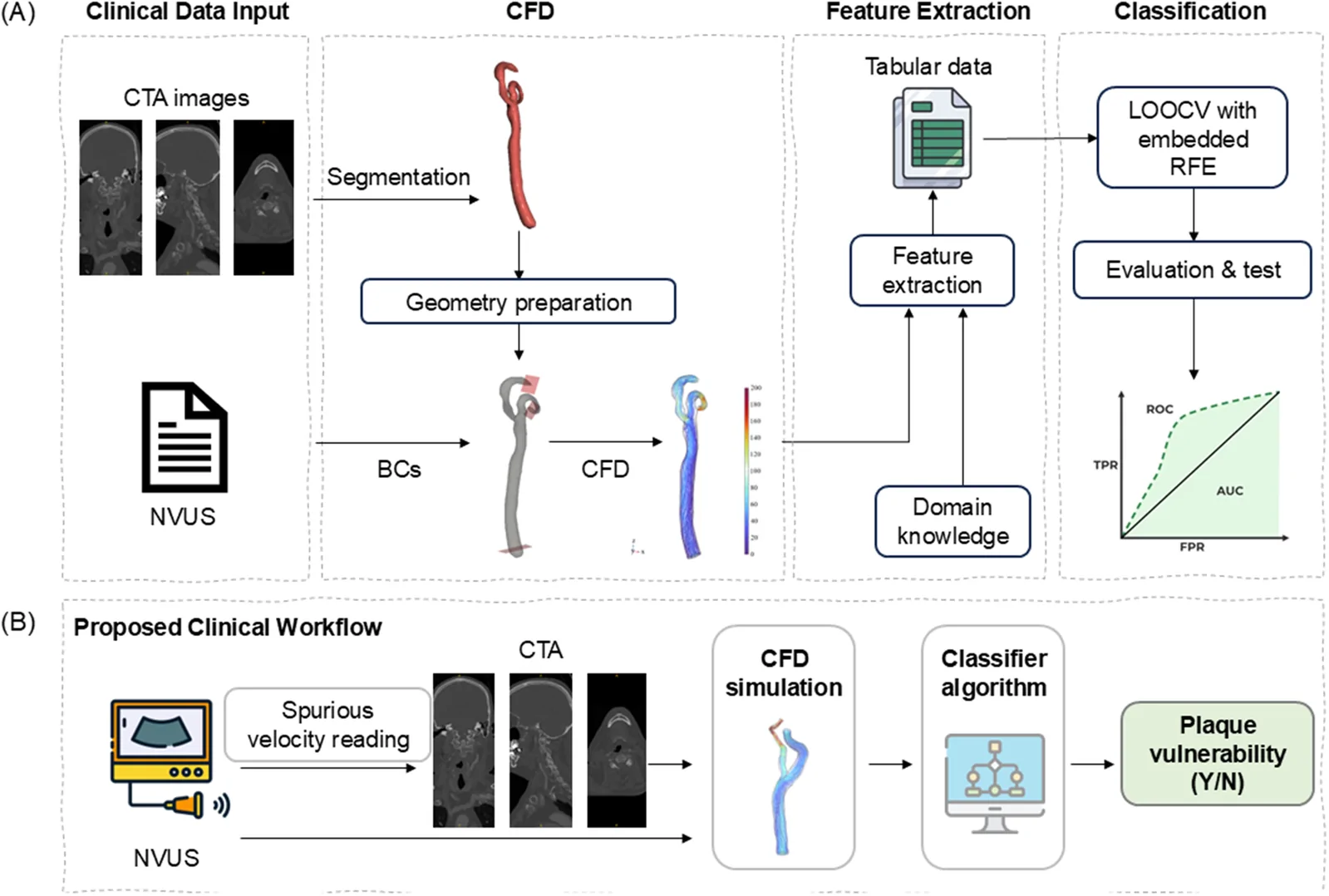
Illustration of (A) the model development process and (B) the potential clinical workflow.

Simulations were performed on a commercially available desktop computer with an Intel i-7-10700F CPU @2.90GHz, featuring 8 cores (16 threads) and 64GB of random-access memory. The multifrontal massively parallel sparse direct solver (MUMPS) in COMSOL was used to solve the system with a relative tolerance of ${\text{1}\text{0}}^{-\text{8}}$.

### 2.5 Data reduction

After acquiring the velocity fields for each carotid (Fig 3A) at peak systole and diastole, geometrical and hemodynamic features were extracted for further analysis. Several key geometrical reference points were defined first. The centerline was extracted using the Vascular Modelling Toolkit (VMTK, v1.4) [44]. To identify the bifurcation point, all centerline points were connected in a graph weighted by pairwise Euclidean distances and the minimum spanning tree was computed. The node with the highest degree in the MST was selected as the bifurcation point, provided its degree was at least three. The three branches were then labeled CCA, ICA and ECA. A spline fit was applied to each branch of the centerlines, which were resampled at uniform intervals of 0.1 mm along its arc length. Owing to the various methods of measuring bifurcation angles reported in previous studies [30,45,46], we measured them systematically from 1mm to 5 mm at 1mm intervals then at 7.5mm, 10 mm, and 20 mm downstream of the bifurcation point, aiming to examine the dependency of bifurcation angles on the measurement location.

**Fig 3:**
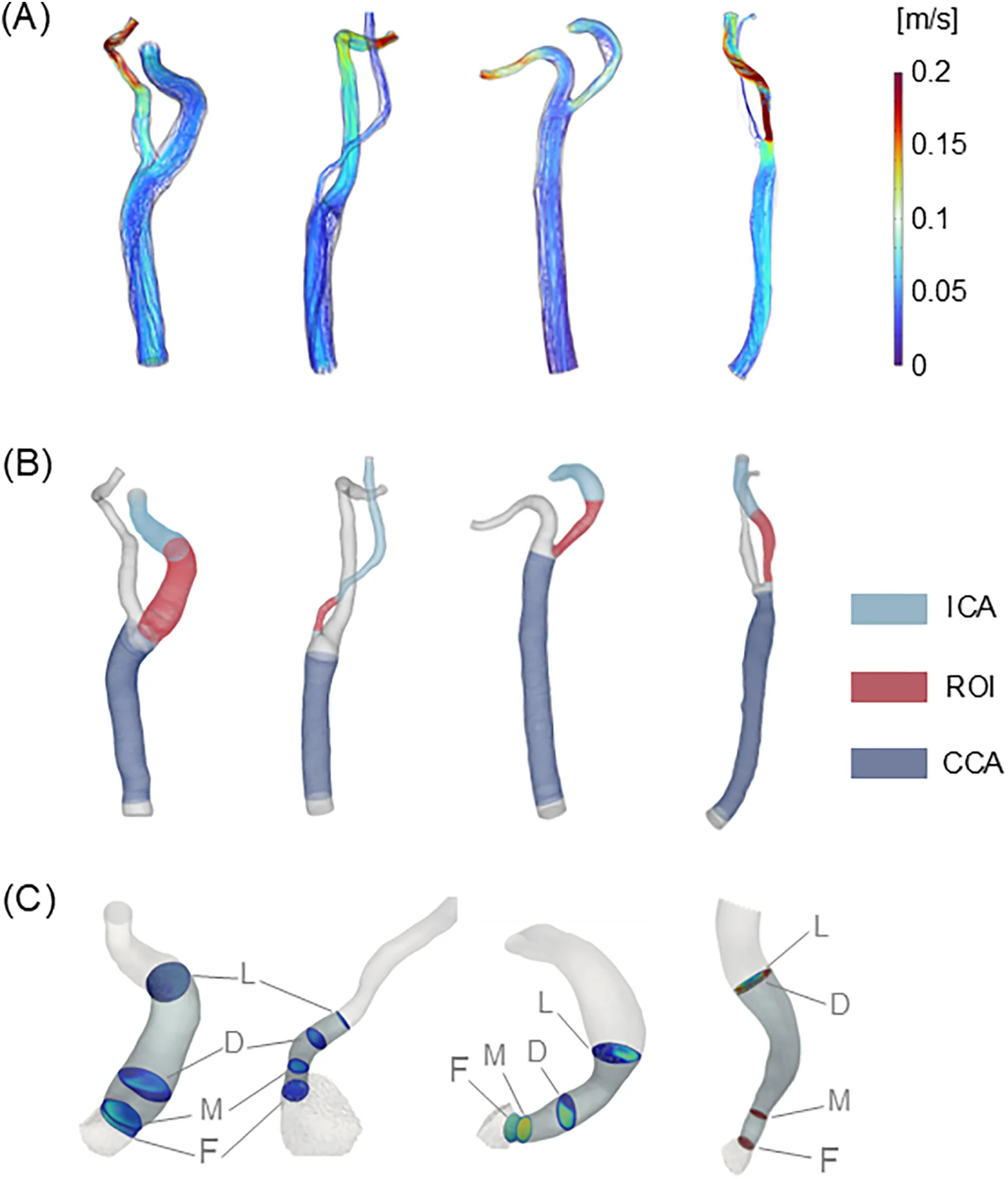
(A) Examples of CFD results with streamlines colored by velocity magnitude. (B) Colored CCA, ICA and ROI. (C) Corresponding ROI with F, M, D, L Planes. The left two columns are non-vulnerable subjects, and the other two are vulnerable.

Along the interpolated centerline point clouds, 2D planes perpendicular to the local tangent were inserted and the intersection with the local vessel wall was extracted. With that done in the ICA, the location with the minimum diameter was identified, where a plane (M Plane) was generated perpendicular to the centerline. The distal plane (D Plane) corresponds to the location where the diameter of the ICA stabilizes beyond the stenotic region, defined by the first zero-crossing of the first derivative of diameter after the M Plane, where post-stenotic dilatation is recovered. A stenotic ratio was defined as $\text{1}-D_{M\;Plane}/D_{D\;Plane}$. This is similar to the method used in NASCET [7], although the values should not be interpreted identically due to the lack of a quantitative definition in NASCET.

The region of interest (ROI) was defined from 5 mm upstream of the M Plane to the reattachment length downstream of the M Plane. The reattachment length is defined by the step height, approximated by ${(D}_{DPlane}-D_{MPlane})/\text{2}$, and a multiplier, which is a function of the local Reynolds number $Re_{D}$. To determine the multiplier, the previous work from Gach et al. [47] was referenced. For Reynolds numbers below 300, the multiplier is linearly related to the Reynolds number as 0.086Re. In the range $\text{3}\text{0}\text{0}\leq Re_{D}\leq \text{1}\text{5}\text{0}\text{0}$, the multiplier maximizes at a constant value of 24. For $Re_{D}>\text{1}\text{5}\text{0}\text{0}$, the multiplier follows an inverse power-law of $\text{5}\text{5}\text{6}\text{1}\text{5}Re^{-\text{1}.\text{0}\text{7}}$. These equations were used to define the disturbed flow region downstream [47], ensuring that all flow dynamics in the stenotic region were captured. The first and last planes of the ROI were designated as the F Plane and the L Plane, respectively (Fig 3C).

Hemodynamic features, including velocity, vorticity, helicity (computed as the local normalized helicity), WSS and their derived statistics, were extracted from each velocity field. Both non-robust measures (e.g., mean, standard deviation, and coefficient of variation) and robust measures (e.g., median, quantiles, and median absolute deviation or MAD) were included. Further, higher order statistical moments of the hemodynamic features (e.g., skewness and kurtosis) were investigated to characterize their symmetry and tailedness. Given the current evidence relating irregular luminal geometry with the presence of surface calcification, lipid-rich necrotic core, and intra-plaque hemorrhage [48–50] and disturbed flow with plaque progression [8,30,51], we hypothesized that intravascular hemodynamics with their higher order statistics may offer more robust measures of overall flow topologies and constitute more effective descriptors to triage patients with vulnerable plaques.

Because individual velocity measurements were used to set the BCs, direct comparison of WSS magnitudes may be misleading. Therefore, a relative WSS was also calculated by normalizing the actual WSS magnitude with a characteristic WSS, computed as $WSS_{char}=\text{4}\mu U/D$ based on the Poiseuille flow assumption, where μ is the dynamic viscosity, U is the peak velocity, and D is the diameter.

For vorticity, the typical vorticity magnitude and its statistical measures were calculated. Additionally, streamwise vorticity was computed as the dot product of the vorticity vector and the tangential vector of the centerline, providing insights on the organization of vortical structures. The distribution of streamwise vorticity indicates the disturbance level of the flow. Statistical analyses were performed using the two-sided nonparametric Mann–Whitney U test, with a p-value less than 0.05 considered statistically significant.

### 2.6 Risk classification models

After calculating the geometrical and hemodynamic features at peak systole and diastole, all features were concatenated for each subject to build the risk classifier. Since the objective of this study was to evaluate the predictive value of geometrical and hemodynamic features, clinical records were excluded. This is justified by (i) the fact that carotid atherosclerotic stenosis affects each side independently and intervention management is determined for each carotid artery separately, and (ii) our target outcome is the plaque vulnerability of each individual carotid. Including clinical data at patient level into the workflow will be appropriate when patient-level outcome, such as the modified Rankin Scale or incidence of cardiovascular event, is considered.

Before training the classifier, the Pearson correlation coefficient matrix between all feature pairs was calculated. For any pair with a Pearson r > 0.8 or r < -0.8, the feature with the higher p-value in univariate comparison was excluded from subsequent analysis. If both features in a pair had identical p-values, the one with the lower variance was removed. These steps guaranteed no collinear variables were passed on to the training/testing routine. Given that univariate comparisons may overlook nonlinear relationships between features and yield misleading significance in small samples, we employed multivariate classifiers to better evaluate the data.

To continue, logistic regression (LR, scikit-learn, v1.2.2) was initially chosen as a baseline classifier because it provides a transparent mapping between features and outcome probability, allowing direct estimation of odds ratio (OR) and straightforward interpretation of the direction and magnitude of each feature’s association with plaque vulnerability.

To mitigate overfitting associated with small datasets, several measures were considered. First, we applied Leave-One-Out Cross-Validation (LOOCV) during training, as implemented in the scikit-learn (v1.2.2) package. The approach iteratively trained the classifier on all but one subject and tested it on the held-out subject. Predicted probabilities of the held-out subjects were pooled across all folds, and the area under the receiver operating characteristic curve (AUC) was computed from the pooled predictions. Second, recursive feature elimination (RFE, scikit-learn, v1.2.2) was embedded within each fold of the LOOCV framework during training. This ensured that the most relevant features were selected from the training data in a specific fold, preventing data leakage across folds. Third, the LR implementation incorporated z-score normalization on the training data and class weight balancing based on class frequency in each fold. Finally, the loss function included an L2 penalty term as regularization, defined as:


$$\begin{array}{c} L=-\sum_{i=\text{1}}^{n}\left[y_{i}log(p_{i})+(\text{1}-y_{i})log(\text{1}-p_{i})\right]+\lambda \sum_{j=\text{1}}^{m}\left(\beta_{j}^{\text{2}}\right) \end{array}$$

where n is the number of samples, m is the number of features, $y_{i}$is the true label for sample i, $p_{i}$ is the predicted probability, $\beta_{j}$ is the model coefficient, and λ is the regularization strength, which was set to 1 in this study.

To test whether a margin-based classifier could capture similar multivariate patterns beyond the log-odds assumption of the LR classifier, we implemented a support vector machine (SVM) with linear kernel and the same routine of LOOCV with embedded RFE to complement our analysis. The linear SVM also maintains interpretability through its weight vector and offers improved generalization in cases where the number of features exceeds the number of samples. The objective function with penalty term is defined as


$$\begin{array}{c} \underset{w,b,\epsilon }{\text{min}}\frac{\text{1}}{\text{2}}{\left\|w\right\|}^{\text{2}}+c\sum_{i}\epsilon_{i} \end{array}$$

subject to $y_{i}\left(w^{T}x_{i}+b\right)\geq \text{1}-\epsilon_{i}$

where w is the weight vector, referred to as feature coefficient in subsequent discussions, $x_{i}$ is the feature vector of the i-th sample, b is the bias, $\epsilon_{i}$ is the slack variable measuring how much the margin is violated, and c is the regularization parameter, which was taken as 1 in this study. With both classifiers trained and tested with same testing routine, the consistency of the results was assessed, especially regarding the most descriptive features.

Since the number of features exceeds the number of samples in this study by far, a dependency study was conducted by iteratively reducing the number of features from the full set down to a single feature, recording the corresponding AUC at each step. The LOOCV and RFE were performed within each iteration independently to avoid data leakage. The final number of features (n*) corresponded to the best AUC, where n* is limited by the number of samples (n* ≤ n). As such, we further minimized the risk of overfitting.

After selecting n*, both LR and SVM classifiers were evaluated with the same LOOCV routine with embedded RFE in each fold. Confusion matrices of the predictions were calculated, along with Brier score, sensitivity, specificity, F1 score, and Cohen’s Kappa. Thresholds were varied to evaluate the tunability of the model. Bootstrapping (n = 2000) was performed on the predictions to give 95%CI of the ROC-AUC and precision-recall (PR) AUC.

Final features were selected independently in each LOOCV fold, and the frequencies were counted to assess the generalizability of the features. Only features selected by at least 12 out of 18 folds were taken for subsequent analysis.

For the LR classifier, mean ORs were computed for each selected feature, and the 95% confidence interval (CI) was estimated using a normal approximation to the log(OR) distribution. ORs greater than one indicate features associated with increased odds of the outcome (vulnerable plaque), while values less than one suggest protective or inverse associations.

For the SVM classifier, mean feature coefficients (95%CI) were calculated using the same normal approximation. Coefficients larger than zero are associated with higher likelihood of the positive class (vulnerable plaque), and vice versa.

These complementary approaches allow evaluation of both probabilistic (LR) and margin-based (SVM) linear classifiers under controlled evaluation routine, providing insight into the stability and discriminative relevance of geometric and hemodynamic features despite the small sample size. We reiterate that it is not our goal to train a classifier ready to be used but rather explore the feasibility of our approach and evaluate whether statistical intravascular hemodynamic features constitute reliable predictors for vulnerable plaques.

## 3. Results

Following the patient selection flow chart (Fig 4), a total of nine patients (18 carotid arteries) were included in the study. Patient characteristics are given in Table 2. Steady-state CFD results were acquired for both peak systolic and diastolic phases. The median (IQR) Reynolds number for all cases and phases was 288 (119–574). Simulation time was generally under one hour per carotid.

**Table 2:** Patient characteristics.

Patient-level Characteristics	
Total patient included (n)	9
Female (n, %)	3, 33%
Age (median [IQR], years)	80 [68, 85]
Smoker (n, %)	3, 33%
Hypertension (n, %)	8, 89%
Dyslipidemia (n, %)	7, 78%
Diabetes/Pre-Diabetes (n, %)	4, 44%
**Vessel-level Characteristics**	
Total carotid included (n)	18
Symptomatic (n, %)	7, 39%
Vulnerable (n, %)	9, 50%
Stenosis severity (NASCET)	
0 (no stenosis)	4, 22%
<50% (mild stenosis)	3, 17%
50%-70% (moderate stenosis)	3, 17%
>70% (severe stenosis)	8, 44%

**Fig 4:**
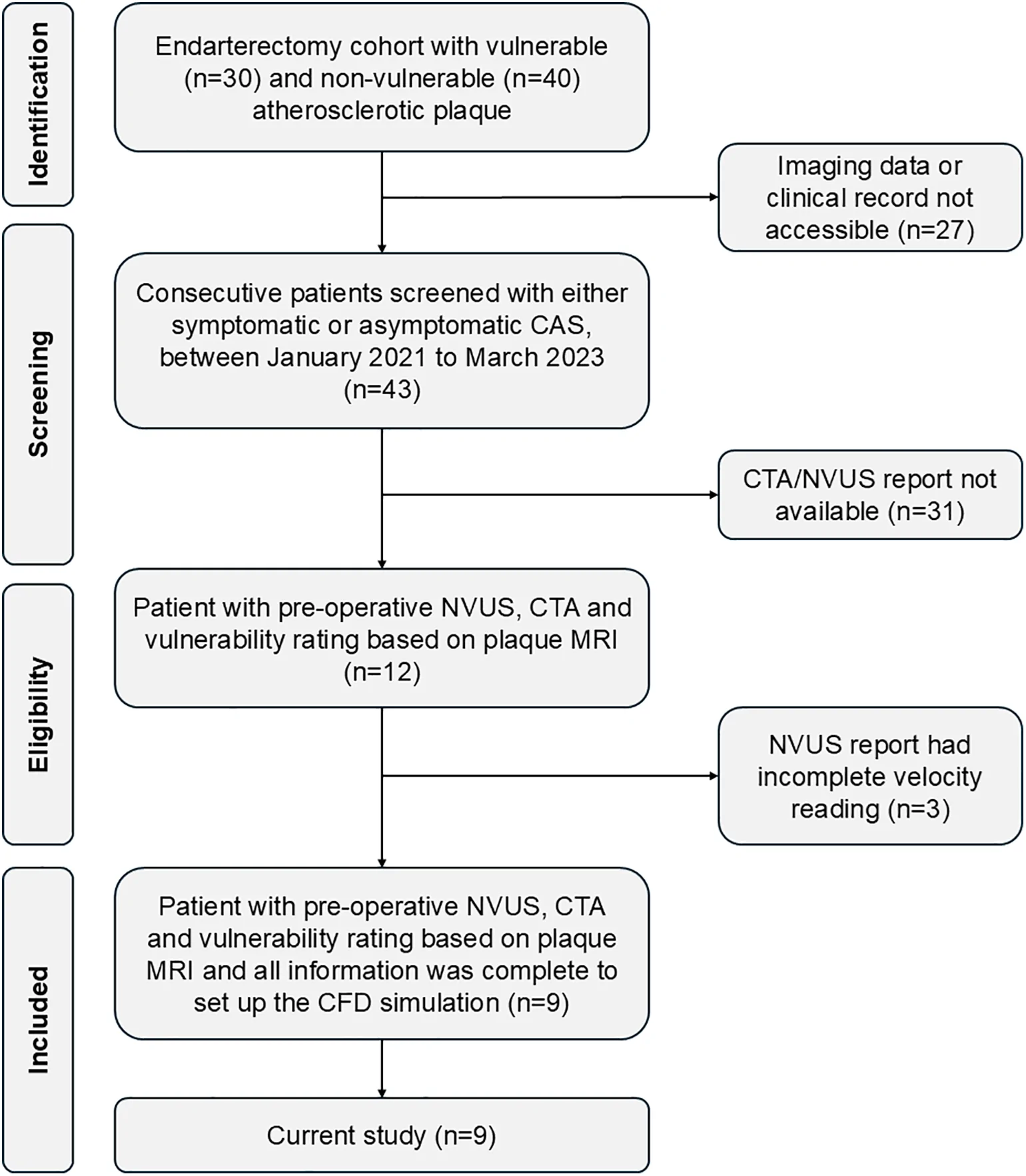
Patient selection flow chart.

The bifurcation angles measured at eight locations downstream of the bifurcation point showed a monotonically decreasing median bifurcation angle (Fig 5A) and all neighboring measurements beyond 5mm were significantly different (p < 0.05). When stratified into vulnerable and non-vulnerable groups (Fig 5B-5I), however, the inter-group comparisons showed no significant difference, but p-values decreased from 1mm to 3mm measurements before increasing (Table 3). Most discriminative measurements were found at 3mm and 4mm after the bifurcation point. No other geometrical features differed significantly between the two groups. A summary of all geometrical features is provided in Table 3.

**Table 3:** Summary of geometrical features (median [IQR]) with p-values.

	Non-vulnerable	Vulnerable	p-value
Stenotic Ratio	0.14 [0.11, 0.34]	0.30 [0.24, 0.37]	0.185
Bifurcation Angle [°], measured at			
1mm downstream of bifurcation point	121 [103, 130]	102 [97, 106]	0.251
2mm downstream of bifurcation point	116 [96, 121]	96 [93, 103]	0.185
3mm downstream of bifurcation point	110 [89, 113]	89 [87, 99]	0.112
4mm downstream of bifurcation point	98 [85, 106]	83 [82, 88]	0.112
5mm downstream of bifurcation point	88 [79, 100]	78 [75, 83]	0.133
7.5mm downstream of bifurcation point	75 [64, 82]	66 [59, 72]	0.158
10mm downstream of bifurcation point	58 [57, 76]	59 [46, 65]	0.377
20mm downstream of bifurcation point	39 [29, 47]	30 [25, 31]	0.480
Mean Diameter ICA [mm]	5.1 [4.0, 5.6]	4.0 [3.5, 4.9]	0.185
Mean Diameter CCA [mm]	6.4 [5.9, 7.2]	7.4 [5.7, 7.7]	0.536
Min Diameter ICA [mm]	4.0 [3.3, 4.6]	3.3 [1.9, 3.9]	0.158
Min Diameter ICA/Mean Diameter ICA	0.85 [0.79, 0.98]	0.72 [0.64, 0.81]	0.133
Mean Diameter ICA/Mean Diameter CCA	0.78 [0.57, 0.80]	0.62 [0.51, 0.70]	0.112
Tortuosity ROI	1.1 [1.0, 1.1]	1.1 [1.0, 1.1]	0.791
Step Height Approximation [mm]	0.43 [0.11, 0.83]	0.52 [0.37, 1.1]	0.251
Reattachment Length [mm]	10 [2.1, 20]	13 [7.2, 19]	0.537
Distance Bifurcation to ICA Min. Plane [mm]	2.7 [2.0, 3.0]	7.9 [2.9, 16]	0.112
Distance ICA Min. Plane to Distal Plane [mm]	4.9 [4.1, 6.2]	6.1 [4.3, 9.1]	0.377
Distance Bifurcation to ROI Last Plane [mm]	14 [8.0, 21]	22 [20, 27]	0.064

**Fig 5:**
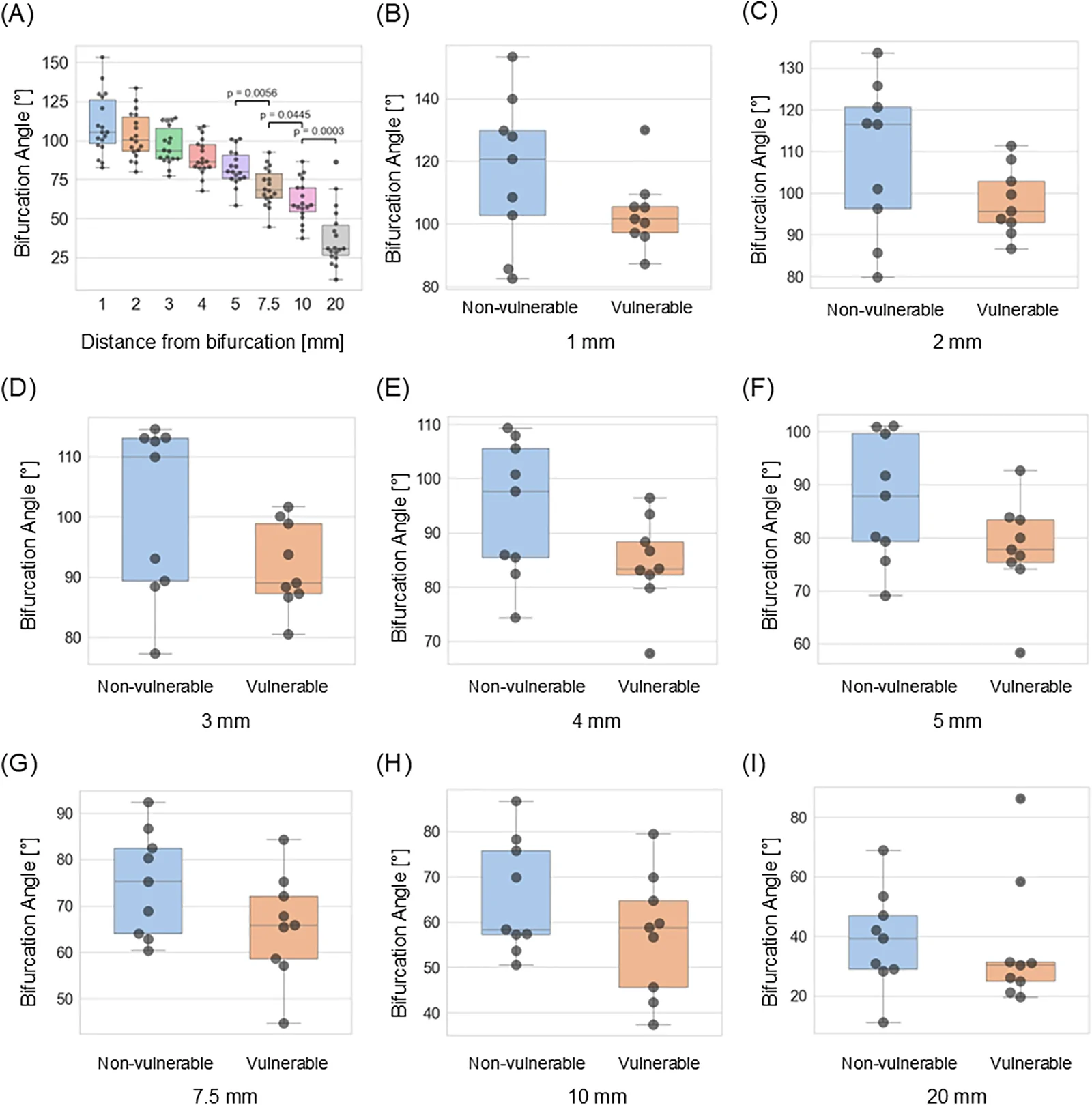
(A) Bifurcation angles measured at various distances downstream of the bifurcation point. (B-I) Comparisons of bifurcation angles between non-vulnerable and vulnerable groups at each location measured from the bifurcation point.

Hemodynamic features with p < 0.05 at peak systole and diastole are given in Table 4. Full lists of features with p < 0.1 are given in the S1 Appendix (Tables C and D). Notably, statistical descriptors of streamwise vorticity and helicity differed significantly between vulnerable and non-vulnerable groups during both peak systole and diastole.

**Table 4:** Summary of hemodynamic features (median [IQR]) at peak systole and diastole with p < 0.05 between vulnerable and non-vulnerable groups. Complete lists of features with p < 0.1 are given in the S1 Appendix (Tables C and D).

Peak systole	Non-vulnerable	Vulnerable	p-value
**M Plane**			
Kurtosis Velocity	-0.54 [-1.4, -0.24]	0.44 [-0.92, 0.90]	0.034
Median Velocity [m/s]	0.35 [0.24, 0.41]	0.89 [0.41, 1.1]	0.042
Mean Helicity	0.12 [0.068, 0.17]	0.013 [-0.023, 0.019]	0.034
Median Helicity	0.070 [0.017, 0.15]	0.0026 [-0.0038, 0.0042]	0.034
**D Plane**			
Kurtosis Helicity	-0.76 [-0.80, -0.66]	-1.0 [-1.1, -0.93]	0.013
**L Plane**			
Mean Streamwise Vorticity [1/s]	0.0044 [-0.00010, 0.0093]	-0.016 [-0.042, 0.0013]	0.027
Skewness Streamwise Vorticity	-0.38 [-1.6, -0.076]	0.74 [0.46, 1.5]	0.010
Median Streamwise Vorticity [1/s]	0.013 [0.011, 0.099]	-0.047 [-0.055, -0.015]	0.017
**ROI**			
Skewness Streamwise Vorticity	6.1 [1.8, 7.8]	11 [6.6, 13]	0.027
**Diastole**	**Non-vulnerable**	**Vulnerable**	**p-value**
**F Plane**			
Kurtosis Velocity	-1.1 [-1.2, -1.1]	-0.82 [-1.1, -0.67]	0.042
MAD Streamwise Vorticity [1/s]	0.016 [0.012, 0.028]	0.033 [0.025, 0.10]	0.034
**M Plane**			
MAD Streamwise Vorticity [1/s]	0.027 [0.021, 0.044]	0.074 [0.049, 0.26]	0.002
Kurtosis Vorticity	0.15 [-0.26, 3.0]	-0.73 [-0.84, -0.63]	0.042
Median Vorticity [1/s]	0.11 [0.082, 0.14]	0.17 [0.13, 0.49]	0.013
**D Plane**			
MAD Streamwise Vorticity [1/s]	0.018 [0.010, 0.035]	0.057 [0.019, 0.086]	0.034
Median Helicity	0.030 [-0.035, 0.10]	-0.051 [-0.086, 0.016]	0.034
**ROI**			
Mean Helicity	0.067 [0.00020, 0.15]	-0.0014 [-0.066, 0.0094]	0.034
Skewness Helicity	-0.22 [-0.28, -0.14]	0.038 [-0.031, 0.062]	0.027

Following the concatenation of geometric and hemodynamic features, each subject was represented by a total of 289 features. Pairwise Pearson correlations among these features are illustrated in Fig 6A, with their distribution shown in Fig 6B. After removing highly correlated features using a threshold of 0.8, 86 features were retained for subsequent analysis (Fig 6C). From the dependency studies, optimal number of features were selected at n* = 10 and n* = 12 for the LR (Fig 7A) and SVM (Fig 7D) classifier, respectively. Confusion matrices for the corresponding model with thresholds at baseline (0.5) and the optimal threshold are shown in Figs 7B–7C and 7E–7F, respectively. Other statistical measures with bootstrapped 95%CI are given in Table 5 for each classifier and each threshold. Individual curves for ROC-AUC, PR-AUC, and the Brier calibration are given in the S1 Appendix (Fig A). In general, both classifiers yielded similar discrimination and calibration, although the SVM demonstrated marginally higher AUCs and slightly better calibration performance. Complementary patient-level results for the LR classifier are given for completeness in the S1 Appendix (Fig B).

**Table 5:** Metrics for LR and SVM classifiers with baseline and optimal thresholds.

	ROC-AUC (95%CI)	PR-AUC (95%CI)	Brier score	Threshold	Sensitivity	Specificity	F1	κ
**LR**	0.77 (0.49 – 0.96)	0.80 (0.52 – 0.98)	0.21	0.5	0.56	0.78	0.63	0.33
				0.4	0.78	0.78	0.78	0.56
**SVM**	0.78 (0.51 – 0.98)	0.85 (0.62 – 1.00)	0.20	0.5	0.78	0.67	0.74	0.44
				0.57	0.78	0.78	0.78	0.56

**Fig 6:**
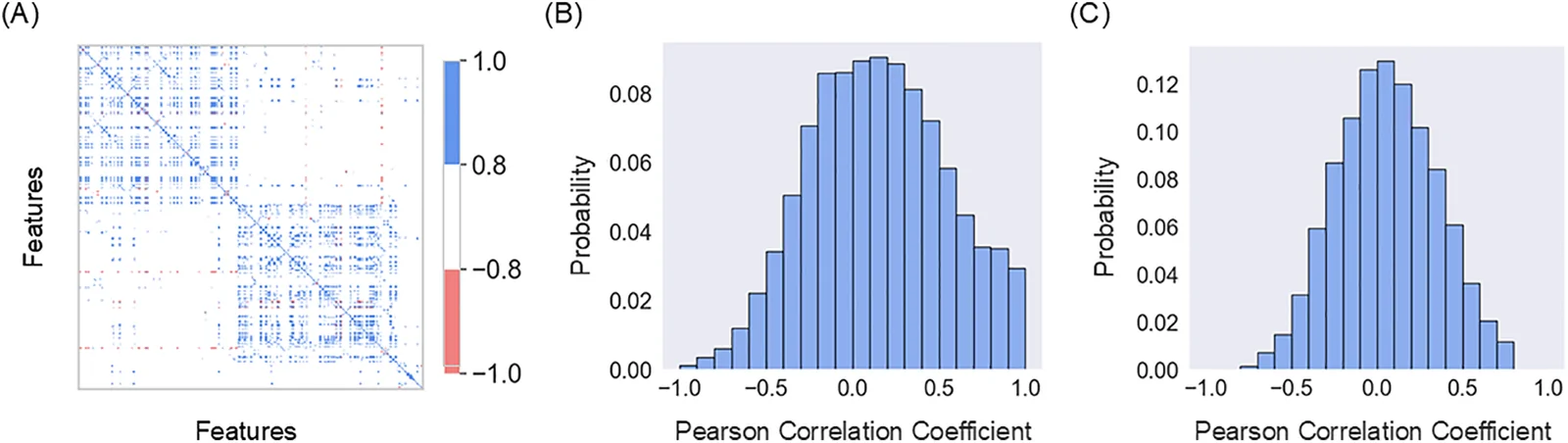
(A) Correlation matrix (Pearson’s r) of all features, highlighting strong correlations (|r|>0.8) in colors. (B) Distribution of correlation coefficient before and (C) after removing highly correlated features.

**Fig 7:**
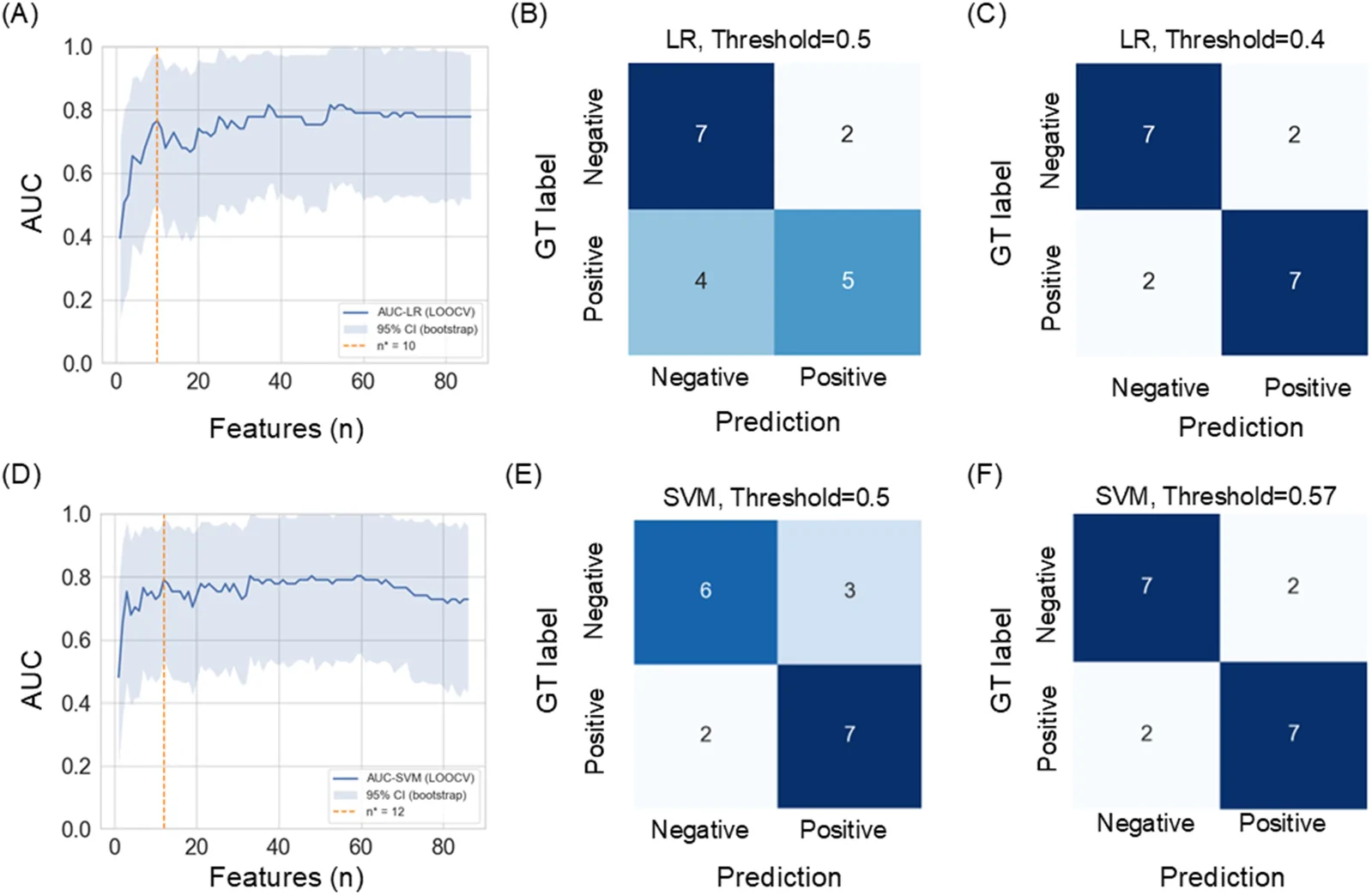
Dependency studies for (A) LR and (D) SVM classifiers over number of features. Shaded area gives the 95%CI. Vertical lines show the feature number (n*) corresponding to the maximum AUC with n smaller than the sample size (n < 18). Confusion matrices for the LR (B, C) and SVM (E, F) classifiers are given with baseline threshold of 0.5 (B, E) and the optimal threshold found for each classifier **(C, F)**.

The selected features for the LR and SVM classifiers are summarized in Fig 8. Features selected in at least 12 out of 18 folds were considered stable, and features selected less frequently were not considered further. It is reassuring that eight features appeared in both classifiers yielded comparable associations with the positive outcome. Notably, mean helicity in the ROI was identified as the most protective feature by both classifiers. Higher order statistical moments of helicity and streamwise vorticity were frequently selected as key predictors. Skewness of streamwise vorticity and helicity at peak systole were among the strongest predictors, while features with Kurtosis of helicity were protective. The only WSS-related feature was the skewness of relative WSS on the surface of the ROI during diastole selected by the LR classifier (mean OR=0.57, 95%CI: 0.50 – 0.64). None of the other measures of WSS magnitude or relative WSS were selected as important contributors by the classifier.

**Fig 8:**
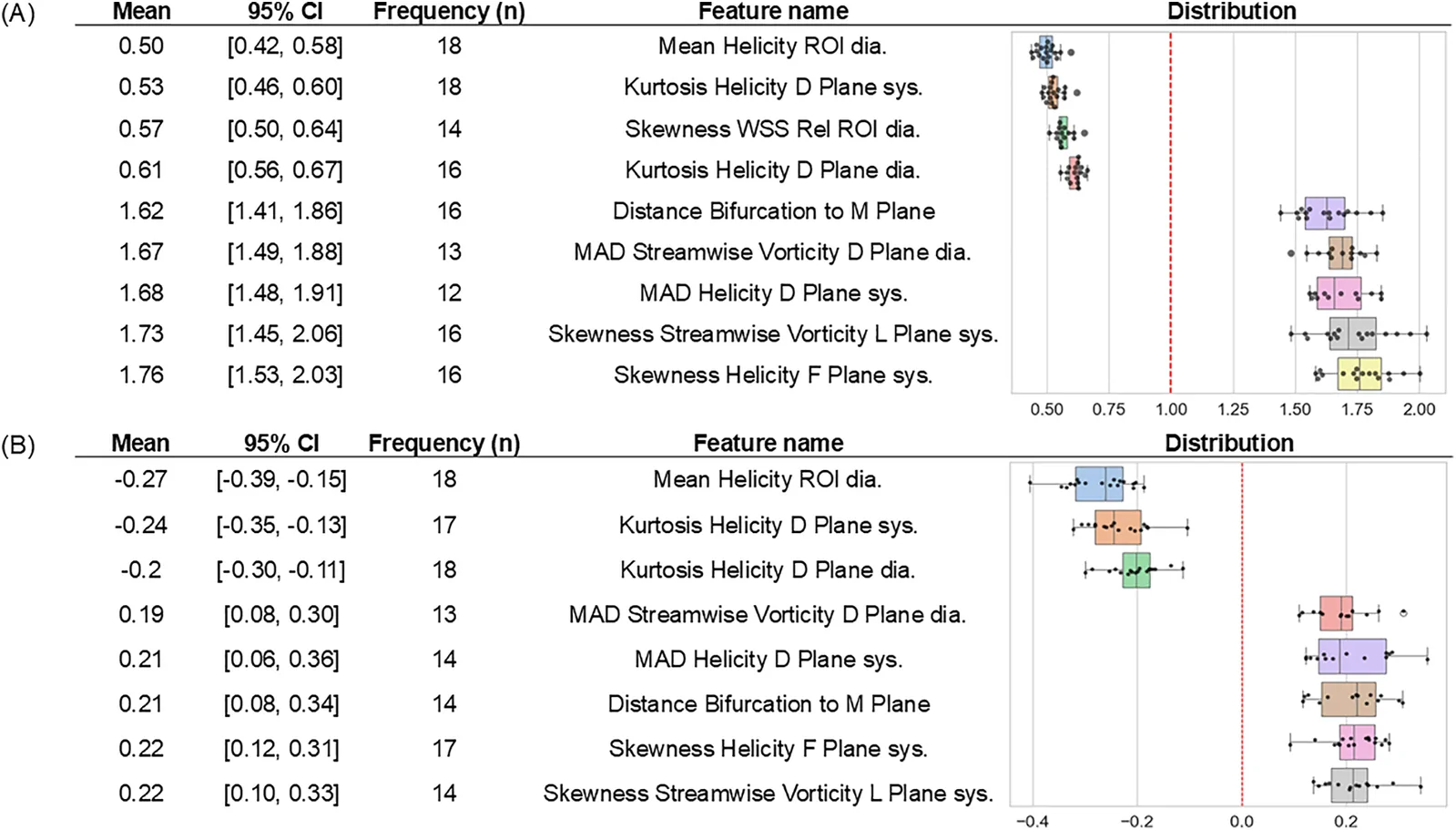
Selected features across all LOOCV folds from the LR (A) and SVM (B) classifiers. The mean and 95% confidence interval (CI) of the Odds Ratios and SVM feature weights are given. Frequency (n) shows the total count a feature was selected across all LOOCV folds (n = 18). The red dashed lines at OR = 1 or feature coefficient = 0 denote no effect. Peak systole: sys. Diastole: dia.

Finally, the distributions of the features selected by the two classifiers, stratified by non-vulnerable and vulnerable groups, are presented in Fig 9. Features with negative (OR<1 or feature coefficient<0) association show a lower median in the vulnerable group (Fig 9A), and vice versa (Fig 9B). These distributions help elucidate the actual physics associated with each feature, which are discussed later in the manuscript.

**Fig 9:**
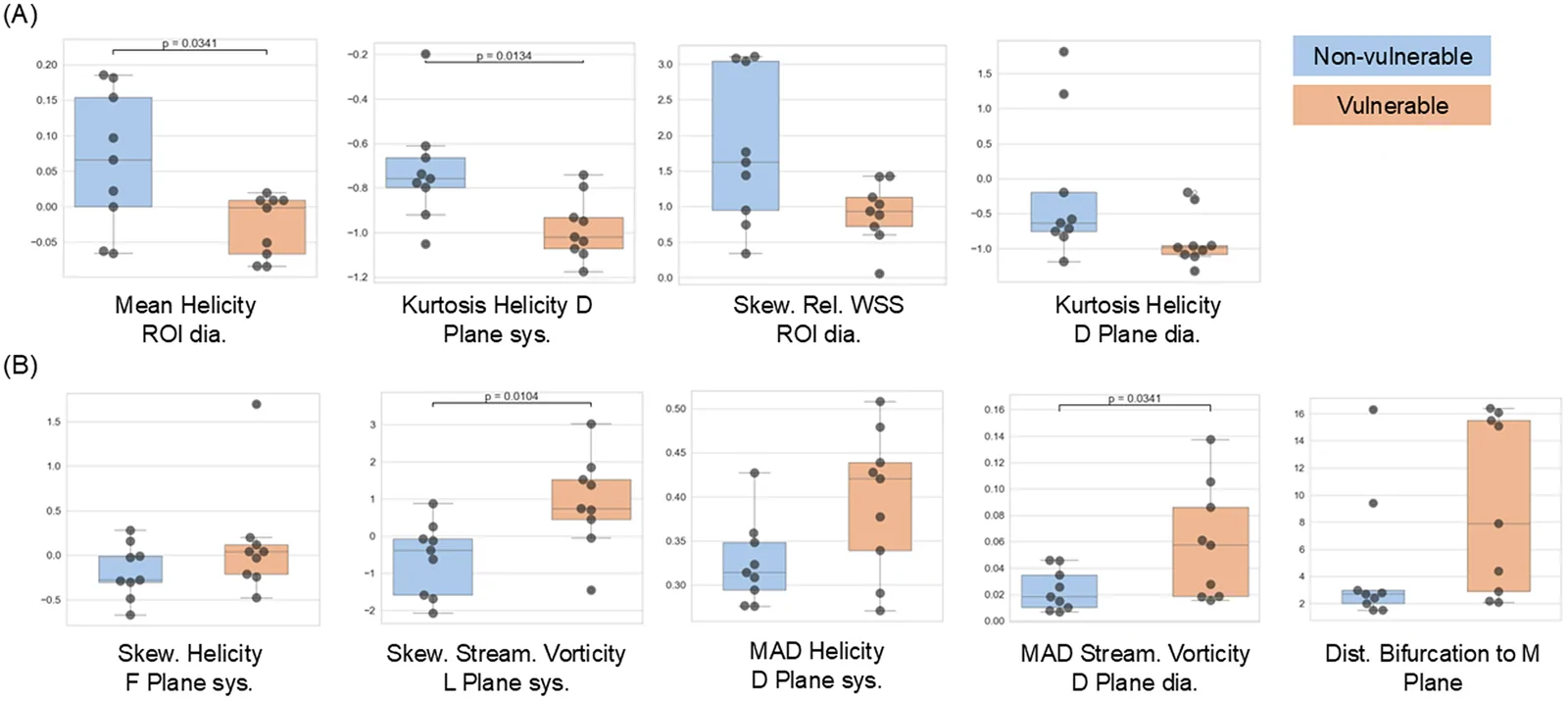
Distributions of features (A) negatively (OR<1 or feature coefficient<0) and (B) positively (OR>1 or feature coefficient>0) associated with the outcome.

## 4. Discussion

### 4.1 Geometrical features

Previous studies have investigated various geometric risk factors for carotid atherosclerosis, but results have been inconsistent. Carotid tortuosity has been reported as both positive [46,52] and negative [24,30] risk predictors, associated with disturbed flow or plaque progression. Diameter metrics, including the ICA diameter [45] and the ICA/CCA diameter ratio [24,25,52] have shown various associations with elevated risk across different cohorts. The bifurcation angle has been reported as a positive predictor of higher plaque volume and progression [45,46], whereas some other studies found no association [30,52].

These inconsistent findings may be attributed to methodological differences in geometric measurements. For example, the bifurcation angle has been measured either between branches at the apex [46], between centerline points located one radius into the ICA/ECA branches [30,45], or using tangential lines of the first 10 mm of the vessel wall from the apex [25,52]. Our results demonstrate that bifurcation angle measurements may differ significantly depending on the chosen reference (Fig 5A), most likely due to the inherent anatomical curvature of the ICA/ECA branches (Figs 1B and 3). In our study, none of the measurements were consistently associated with plaque risk. Bifurcation angle measured at 4mm was selected in the LR classifier once out of 18 folds, so it was not included in our analysis. From a clinical perspective, longitudinal measurements of the bifurcation angle might be more informative as a surrogate feature for plaque growth.

Other previously reported risk factors, including tortuosity, ICA diameter, and the ICA/CCA diameter ratio, showed no significant differences in our univariate analysis (Table 3), nor were they selected as important features in our classifiers (Fig 8). It might suggest that many of the previously suggested features either lack unified definitions or are not generalizable. In this regard, using data-driven methods to identify the most stable/generalizable features might be a good first step for subsequent clinical studies. These results underscore (i) the crucial importance of standardized geometric definitions for reproducible risk assessment and (ii) the need to investigate whether a shift towards (intra-)vascular hemodynamic biomarkers provides more effective measures for clinical assessment.

### 4.2 Hemodynamic features

WSS and its various derived forms have been considered important hemodynamic features for predicting adverse events in CAS patients [33]. The underlying rationales were twofold. First, excessive WSS exerted on the plaque surface may exceed a critical stress level and cause rupture [35,53–55]. Second, lower or oscillating WSS may promote plaque progression [23,32,34]. However, a mechanistic consensus remains elusive.

From a technical perspective, WSS quantification is highly sensitive to geometric processing and near-wall velocity, whose fidelity depends on imaging resolution [56], surface reconstruction methods [57], and the treatment of near-wall velocity fields [58]. While 4DMR-derived WSS showed a 15% deviation from theoretical values in a simple straight tube phantom [59], severe stenotic cases demonstrated discrepancies of up to 38% in the averaged WSS [60]. These uncertainties compound in further derived features, such as WSS topological skeletons [32], limiting their clinical relevance.

The current study proposed several methodological improvements to address these limitations. First and foremost, we investigated intravascular hemodynamic features, including helicity and streamwise vorticity. A few studies in coronary artery disease have reported that intravascular fluid quantities such as helicity are less sensitive to noise, lumen edge definition, and spatial-temporal resolution than WSS assessment [61]. Streamwise vorticity was a novel feature examined in this study, as it not only quantifies the vorticity of the flow but also relates to the organization of the vortical structures. Second, we extracted higher order statistical moments, such as skewness and kurtosis, to characterize the symmetry and tailedness of various features. Last but not least, the steady-state CFD setup incorporated BCs directly measured from NVUS at peak systole and diastole, avoiding uncertainties associated with generic pulsatile waveforms, which could potentially lead to false representations of the flow field [31,33,61] and of all subsequent calculations of hemodynamic features.

Our results revealed that no WSS-related features showed significant differences in the univariate comparison (Table 4), and only the skewness of relative WSS at diastole was selected as a risk factor in the LR classifier (Fig 8). In contrast, intravascular flow features emerged as strong candidates for classification, including helicity and streamwise vorticity. For instance, positive mean helicity within the ROI during diastole (Fig 8, 9) was associated with non-vulnerable plaques. This observation corroborates the current evidence that higher helicity is protective against atherosclerotic plaque progression in coronary artery disease [61,62] and in carotid bifurcation [51].

In addition, the higher order statistical moments provided insights into flow organization. For example, the kurtosis of helicity at the D plane during systole emerged as one of the strongest protective features in both classifiers. Higher kurtosis values (leptokurtic) indicate concentrated helicity distributions, reflecting highly organized helical flow structures, while lower kurtosis values (platykurtic) reflect broader, flatter distributions indicative of disturbed and less coherent flow organization. This relationship between flow organization and plaque risk aligns with published evidence showing that irregular luminal geometry is associated with the presence of surface calcification, lipid-rich necrotic core, and intra-plaque hemorrhage, which are confirmed predictors of elevated plaque risk [50]. As identified by Hokari et al. [48] in 67 carotid stenotic (30–69%) patients, irregular shape of the carotid lumen was the most powerful variable to predict ischemic events, which correlate with plaque rupture. From multi-modal imaging findings in real world data [49], plaque surface irregularity was identified as a risk feature for stroke. These findings motivated our hypothesis that intravascular hemodynamics with their higher order statistics may offer surrogate measures of vessel topologies, where more irregular surfaces lead to more disturbed flow.

These data-driven observations comprise early evidence to support our hypothesis that statistical measures of intravascular hemodynamic features may serve as surrogate markers of luminal geometry regularity, effectively capturing the flow disturbances caused by high-risk plaque components. Our results, upon further validation, seem to underpin a paradigm shift toward intraluminal flow characterization for improved carotid plaque risk stratification.

### 4.3 Clinical relevance and classifier robustness

While the current gold standard involves plaque MRI assessment, which has limited availability, our approach establishes a routine using widely accessible NVUS and CTA imaging. For suspected CAS patients, NVUS is usually done as first-line imaging, and the CTA procedure requires no more than 10 minutes. The CFD analysis utilizes commercially available software requiring minimal expert input once configured, while subsequent data processing and analysis are largely automated. This end-to-end data-driven framework could be easily integrated into current clinical workflows.

In current practice, patients with suspected CAS are often referred to tertiary centers for plaque MRI assessment, which creates bottlenecks due to limited scanner availability and requires specialized radiological expertise. The proposed workflow could serve as a pre-screening step, triaging patients who most likely harbor vulnerable plaques for subsequent plaque MRI, while sparing those with low-risk profiles from unnecessary referrals. This would reduce the demand on tertiary MRI resources, shorten diagnostic delays, and potentially lower per-patient costs.

In this pilot study, the sample size was rather small, so robustness of the results was rigorously tested, and results were carefully interpreted. First, we used LOOCV for cross validation, with embedded RFE performed inside each fold to prevent data leakage. Second, input normalization and class weight balancing were implemented in both LR and SVM classifiers. Third, features were selected by frequency and commonly used metrics were presented for transparency. The test on optimal threshold for the confusion matrix demonstrated the tunability of the classifiers. The threshold can be optimized to reduce false positives so that unnecessary interventions can be avoided, which are potentially harmful for the patient and are associated with increased medical costs. Finally, the selected features were largely consistent from the two classifiers and correlated well with available evidence from engineering and clinical studies, further supporting the validity of our data-driven approach. After validating in a prospective cohort, we believe the proposed workflow will have positive clinical and economic impacts in various clinical scenarios. Formal variance inflation factor analysis was not feasible given the high feature-to-sample ratio; however, pairwise correlation filtering, L2 regularization, and embedded RFE collectively mitigate the effects of residual multicollinearity. The pairwise correlations among the final selected features (Fig E in the S1 Appendix) confirm that no feature pair exceeds the |r| = 0.8 threshold, with most pairs below |r| = 0.5.

The two classifiers achieved ROC–AUC of 0.77 (95%CI: 0.49–0.96) and 0.78 (95%CI: 0.51–0.98), respectively, suggesting moderate discriminative performance for identifying vulnerable carotid plaques. The wide confidence interval reflects substantial statistical uncertainty, at least partially attributable to the small sample size, and these results should therefore be viewed as exploratory rather than definitive.

To further assess robustness, we performed a sub-analysis excluding the four non-stenotic subjects (NASCET = 0%) from the cohort (see Table 2), whose hemodynamic profile may differ from that of arteries harboring stable plaques. Results are presented in the S1 Appendix (Table E and F, Fig C and D). Classifier performance was largely maintained (LR: ROC-AUC = 0.80, PR-AUC = 0.75; SVM: ROC-AUC = 0.71, PR-AUC = 0.86), and, critically, higher order statistics of helicity and streamwise vorticity were consistently identified by both classifiers in both cohorts. This convergence reinforces the robustness of the hemodynamic signal independently of the non-stenotic subjects. Nevertheless, given the further reduced sample size in the sub-cohort, these findings should be interpreted as supportive, rather than confirmatory, of the proposed workflow.

In clinical practice, patients presenting for carotid evaluation are typically assessed bilaterally, and the contralateral artery may be non-stenotic. The proposed workflow would therefore encounter non-stenotic vessels as a matter of routine, and having the ability to correctly classify them, rather than defaulting to a stenotic-only model, could be of practical importance. A stratified approach restricted to plaque-bearing arteries might be of interest in a prospective cohort targeting workflows in tertiary centers to avoid unnecessary surgeries and costs.

In sum, the consistent association of helicity and streamwise vorticity, along with their higher order statistical moments, with plaque vulnerability is encouraging and motivates prospective validation. With larger prospective datasets, the most informative features and their underlying hemodynamic physics could be examined further, informing a refined and more generalizable feature set for future classifiers.

### 4.4 Limitations

This pilot study served primarily as a feasibility proof-of-concept for the proposed workflow. Although the robustness of our classifiers was carefully tested, the limited sample size prevents generalizable claims about classifier performance or definitive interpretation of the selected features. The conclusions should therefore be viewed as hypothesis generating rather than confirmatory. Key limitations are outlined below, and recommendations are briefly discussed to inform further investigations into the topic.

At the study level, the retrospective design and single-centric data collection introduce potential selection and protocol bias because the data were not originally collected for the specific purpose of the present analysis. A larger, multicentric, prospective cohort with refined selection criteria accounting for baseline characteristics, such as time delay between tests, antithrombotic treatment, and comorbidities is needed to mitigate these biases. The current cohort comprised exclusively patients referred for carotid endarterectomy, which limits generalizability. Future studies should include patients without an indication for intervention (surgical or endovascular) to better represent the broader clinical population. The clinical relevance of including non-stenotic subjects warrants further consideration in light of realistic referral pathways. A stratified approach restricted to plaque-bearing arteries should be explored in a prospective cohort.

At the patient level, inter-rater variability should be assessed for subjects with complex or borderline imaging findings. A more diverse and detailed stratification according to the AHA Classification guidelines [13] or the Plaque-RADS score [39] may be appropriate as clinical endpoints, allowing more nuanced risk modelling beyond the binary classification used here. Intra-subject dependencies of vessel geometries should be further explored to build patient-level recommendations. This would further enable the incorporation of long-term clinical outcomes such as stroke, transient ischemic attack, myocardial infarction, and death, providing deeper insight into the clinical management of CAS patients.

From a technical perspective, the significance level for univariate comparisons and the effect sizes of classifiers should be further examined. As more imaging data becomes available, a fully automated segmentation model should be trained to extract the vessel geometry without human interaction. A pre-trained nnU-Net [63] or vision transformer would be appropriate for the task. This could be helpful to mitigate the uncertainties associated with human interaction. With that, the definitions of various geometric features could be explored more rigorously to confirm the optimal choice. After that, a consensus statement on the processing steps, from imaging to feature extraction, could be established. Regarding CFD, the choice of steady-state, Newtonian, laminar model was a compromise for simplicity. More sophisticated setups should be explored in future work to discuss the relevance in clinical settings.

From a practical perspective, the use of commercial software might pose financial limitations for some clinics by means of both licensing fees and personnel costs. It would be beneficial to integrate an open-source CFD code, such as OpenFOAM [64], into our workflow to make it more accessible and fully automated. On the classifier, larger, multicentric data should be used to train and test the classifiers and evaluate the statistics further. Hyperparameter tuning will be crucial to produce deploy-ready classifiers. With that, it is our hope that the proposed framework will benefit communities across biomedical engineers to clinical researchers.

## 5. Conclusions

In this pilot study, we showed that by using NVUS records and CTA imaging, we were able to triage patients for plaque vulnerability benchmarked against plaque MRI assessment. Using leave-one-out cross-validation combined with recursive feature elimination, we achieved AUC = 0.77 and AUC = 0.78 for LR and SVM classifiers, respectively. The descriptive power of geometrical features remains elusive, but higher order statistical moments of streamwise vorticity and helicity seem promising as surrogate hemodynamic predictors of plaque vulnerability. Validation in larger, multicentric cohorts will be essential to confirm the generalizability and clinical applicability of these findings.

## Supporting information

S1 Appendix Supporting information. (DOCX)

## Abbreviations

4DMR: four-dimensional magnetic resonance
AUC: area under the receiver operating characteristic curve
BC: boundary condition
CA: carotid artery
CAS: carotid atherosclerotic stenosis
CCA: common carotid artery
CFD: computational fluid dynamics
CI: confidence interval
CTA: computed tomography angiography
DICOM: digital imaging and communications in medicine
ECA: external carotid artery
ICA: internal carotid artery
LOOCV: leave-one-out cross validation
MRI: magnetic resonance imaging
NASCET: North American symptomatic carotid endarterectomy trial
NIFTI: neuroimaging informatics technology initiative
NVUS: neurovascular ultrasound
OR: odds ratio
RFE: recursive feature elimination
ROI: region of interest
WSS: wall shear stress
