# Supplementary material for: Data driven approach to identify vulnerable plaques in carotid atherosclerosis using computed tomography imaging and computational fluid dynamics: A pilot study
Source: PLOS Digit Health. 2026 Oct 7;5(10):e0001762. doi: 10.1371/journal.pdig.0001762 (PMC13645035; doi:10.1371/journal.pdig.0001762)
Supplement: Supplementary file 1 [file pdig.0001762.s001.docx]

**Supporting Information**

**Data driven approach to identify vulnerable plaques in carotid atherosclerosis using Computed Tomography imaging and Computational Fluid Dynamics: a pilot study**

Shaokai Zheng PhD ^1, 2, 3, ¶, *^, David Bervini MD ^4, ¶^, Hugo Besson MSc ^1^, Sofía González Martínez MSc ^1^, Mirjam R Heldner MD ^3^, Mandy Müller MD PhD ^4^, Thomas Meinel MD PhD ^3^, Johannes Kaesmacher MD PhD ^2^, Andreas Harloff MD ^5^, Dominik Obrist PhD ^1^

1 ARTORG Center for Biomedical Engineering Research, University of Bern, Bern, Switzerland

2 Department of Diagnostic and Interventional Neuroradiology, Inselspital, Bern University Hospital and University of Bern, Bern, Switzerland

3 Department of Neurology, Inselspital, Bern University Hospital and University of Bern, Bern, Switzerland

4 Department of Neurosurgery, Inselspital, Bern University Hospital and University of Bern, Bern, Switzerland

5 Department of Neurology and Neurophysiology, Faculty of Medicine, Medical Center-University of Freiburg, Freiburg, Germany

* Corresponding author

Email: [shaokai.zheng@outlook.com](mailto:shaokai.zheng@outlook.com)

^¶^ These authors contributed equally to this work.

**Patient selection**

The original cohort of the study (unpublished at the time of writing) was collected to identify candidate biomarkers of atherosclerotic plaque vulnerability in endarterectomy specimens of patients with carotid disease (ethical approval by the local ethics committee KEK Bern, No.2020-03019). Inclusion criteria included (i) be over 18 years old. (ii) Have extracranial carotid artery stenosis >50% (NASCET or non-invasive equivalent) caused by atherosclerosis. (iii) Scheduled for carotid endarterectomy (CEA). (iv) Have either symptomatic carotid stenosis (focal ischemic symptoms in the carotid territory within the past 6 months — e.g. stroke, TIA, amaurosis fugax, ischemic retinal infarct), or asymptomatic carotid stenosis (no ischemic symptoms in the past 6 months). (v) Be clinically stable and independent at inclusion (modified Rankin Scale < 2). Exclusion criteria included (i) pregnancy (confirmed by urine or blood test). (ii) Inability to understand or comply with study procedures or follow-up. (iii) Contraindications to contrast MRI, including MRI-incompatible pacemaker, MRI-incompatible metal implants, Claustrophobia, or known hypersensitivity/allergy to Gadolinium contrast agent. (iv) Non-atherosclerotic carotid disease (e.g. dissection, fibromuscular dysplasia, post-radiotherapy). (v) Previous CEA or carotid stenting in the same artery. Further details can be found on the swissethics database.

**Imaging protocol**

Technical details of the imaging protocol are given in Table A-B.

Table A. Scanning protocol for MAGNETOM Skyra Fit (Siemens Healthineers, Erlangen, Germany).

Table B. Scanning protocol for MAGNETOM 3.0T XT (Siemens Healthineers, Erlangen, Germany)*.*

Table C. Summary of hemodynamic features (median [IQR]) at peak systole with p<0.1 between vulnerable groups.

| **Peak systole** | **Non-vulnerable** | **Vulnerable** | **p value** |
| --- | --- | --- | --- |
| **M Plane** |  |  |  |
| Mean Velocity [m/s] | 0.38 [0.32, 0.42] | 0.76 [0.38, 0.99] | 0.093 |
| Skewness Velocity | -0.39 [-0.80, 0.10] | -1.3 [-1.4, -0.55] | 0.052 |
| Kurtosis Velocity | -0.54 [-1.4, -0.24] | 0.44 [-0.92, 0.90] | 0.034 |
| Median Velocity [m/s] | 0.35 [0.24, 0.41] | 0.89 [0.41, 1.1] | 0.042 |
| Mean Helicity | 0.12 [0.068, 0.17] | 0.013 [-0.023, 0.019] | 0.034 |
| Median Helicity | 0.070 [0.017, 0.15] | 0.0026 [-0.0038, 0.0042] | 0.034 |
| Kurtosis Vorticity | 2.3 [1.5, 5.4] | 0.099 [-0.11, 1.0] | 0.093 |
| **D Plane** |  |  |  |
| Skewness Streamwise Vorticity | -0.62 [-1.6, 0.089] | 0.32 [-0.69, 2.1] | 0.093 |
| Median Streamwise Vorticity [1/s] | 0.029 [0.020, 0.063] | -0.021 [-0.033, 0.0035] | 0.077 |
| Kurtosis Helicity | -0.76 [-0.80, -0.66] | -1.0 [-1.1, -0.93] | 0.013 |
| MAD Helicity | 0.31 [0.29, 0.35] | 0.42 [0.34, 0.44] | 0.093 |
| **L Plane** |  |  |  |
| Mean Streamwise Vorticity [1/s] | 0.0044 [-0.00010, 0.0093] | -0.016 [-0.042, 0.0013] | 0.027 |
| Skewness Streamwise Vorticity | -0.38 [-1.6, -0.076] | 0.74 [0.46, 1.5] | 0.010 |
| Median Streamwise Vorticity [1/s] | 0.013 [0.011, 0.099] | -0.047 [-0.055, -0.015] | 0.017 |
| **ROI** |  |  |  |
| Skewness Streamwise Vorticity | 6.1 [1.8, 7.8] | 11 [6.6, 13] | 0.027 |

Table D. Summary of hemodynamic features (median [IQR]) at diastole with p<0.1 between vulnerable groups.

| **Diastole** | **Non-vulnerable** | **Vulnerable** | **p value** |
| --- | --- | --- | --- |
| **F Plane** |  |  |  |
| Kurtosis Velocity | -1.1 [-1.2, -1.1] | -0.82 [-1.1, -0.67] | 0.042 |
| MAD Streamwise Vorticity [1/s] | 0.016 [0.012, 0.028] | 0.033 [0.025, 0.10] | 0.034 |
| Mean Helicity | 0.035 [0.0011, 0.064] | -0.018 [-0.029, 0.0044] | 0.064 |
| **M Plane** |  |  |  |
| SD Streamwise Vorticity [1/s] | 0.071 [0.030, 0.10] | 0.13 [0.074, 0.48] | 0.052 |
| MAD Streamwise Vorticity [1/s] | 0.027 [0.021, 0.044] | 0.074 [0.049, 0.26] | 0.002 |
| Median Streamwise Vorticity [1/s] | 0.0022 [0.0017, 0.017] | -0.0013 [-0.0082, 0.0022] | 0.077 |
| Mean Helicity | 0.040 [0.0020, 0.076] | -0.038 [-0.054, 0.0029] | 0.052 |
| Median Helicity | 0.012 [0.0012, 0.065] | -0.0049 [-0.016, -0.0011] | 0.064 |
| Mean Vorticity [1/s] | 0.11 [0.092, 0.21] | 0.20 [0.13, 0.52] | 0.052 |
| Skewness Vorticity | 0.95 [0.77, 1.9] | 0.34 [0.25, 0.71] | 0.077 |
| Kurtosis Vorticity | 0.15 [-0.26, 3.0] | -0.73 [-0.84, -0.63] | 0.042 |
| MAD Vorticity [1/s] | 0.064 [0.043, 0.070] | 0.11 [0.061, 0.34] | 0.070 |
| Median Vorticity [1/s] | 0.11 [0.082, 0.14] | 0.17 [0.13, 0.49] | 0.013 |
| **D Plane** |  |  |  |
| SD Streamwise Vorticity [1/s] | 0.028 [0.018, 0.061] | 0.082 [0.031, 0.24] | 0.052 |
| Skewness Streamwise Vorticity | -0.26 [-0.64, -0.14] | 0.076 [-0.25, 0.64] | 0.093 |
| MAD Streamwise Vorticity [1/s] | 0.018 [0.010, 0.035] | 0.057 [0.019, 0.086] | 0.034 |
| Mean Helicity | 0.060 [-0.065, 0.15] | -0.066 [-0.11, 0.0043] | 0.064 |
| Kurtosis Helicity | -0.63 [-0.75, -0.20] | -0.98 [-1.1, -0.95] | 0.064 |
| MAD Helicity | 0.23 [0.14, 0.25] | 0.27 [0.26, 0.40] | 0.052 |
| Median Helicity | 0.030 [-0.035, 0.10] | -0.051 [-0.086, 0.016] | 0.034 |
| **L Plane** |  |  |  |
| Mean Streamwise Vorticity [1/s] | 0.00090 [0.00050, 0.0021] | -0.0027 [-0.0045, 0.0012] | 0.064 |
| SD Vorticity [1/s] | 0.028 [0.016, 0.080] | 0.084 [0.046, 0.27] | 0.077 |
| MAD Vorticity [1/s] | 0.016 [0.012, 0.046] | 0.062 [0.021, 0.095] | 0.064 |
| **ROI** |  |  |  |
| Skewness WSS Rel | 1.6 [0.95, 3.0] | 0.94 [0.72, 1.1] | 0.052 |
| Mean Helicity | 0.067 [0.00020, 0.15] | -0.0014 [-0.066, 0.0094] | 0.034 |
| Skewness Helicity | -0.22 [-0.28, -0.14] | 0.038 [-0.031, 0.062] | 0.027 |
| Median Helicity | 0.028 [0.00030, 0.091] | -0.00010 [-0.032, 0.0036] | 0.093 |
| Mean Vorticity [1/s] | 0.078 [0.056, 0.094] | 0.15 [0.077, 0.38] | 0.093 |
| MAD Vorticity [1/s] | 0.023 [0.021, 0.038] | 0.062 [0.027, 0.13] | 0.052 |
| Median Vorticity [1/s] | 0.066 [0.046, 0.089] | 0.12 [0.065, 0.26] | 0.077 |

Fig A. ROC-AUC, PR-AUC, and Brier calibration curves for the logistic regression (A-C) and support vector machine (D-F) classifier. These figures are given to complement Fig 7 and Table 5 in the main manuscript.

Fig B. Results from patient-level LOOCV using the logistic regression (LR) classifier, showing (A) dependency study as defined in the main manuscript, (B) ROC-AUC curve, (C) PR-AUC curve, (D) Brier calibration curve, and (E) confusion matrix at threshold=0.4. Slight performance drop is observed in all metrics, given the smaller training sample size per fold.

**Sub-cohort analysis without non-stenotic subjects**

In this section, we present results without non-stenotic (NASCET=0%) subjects (n=4) as a measure of sensitivity analysis for the proposed workflow. For the following analysis, a total of n=14 subjects are included (see Table 2) with subjects ranging from mild stenosis (NASCET <50%) to severe stenosis (NASCET >70%). Of the 14 arteries, 9 were classified as vulnerable and 5 as non-vulnerable. Summaries of geometrical features and hemodynamic features (p<0.1) are given in Table E, F.

After removing highly correlated features using a threshold of 0.8, 70 features were retained for subsequent analysis. From the dependency study of AUC over number of features, the LR classifier yielded optimal performance with only one feature (Fig C), where the Kurtosis of helicity at D Plane during systole (OR:0.24, CI: 0.21-0.28) alone gave ROC-AUC=0.80 and PR-AUC=0.75 (Fig C). While these numbers should be interpreted with caution, the selected feature corroborates the findings of the main study and was selected by both classifiers as one of the most descriptive features. Regarding the SVM classifier (Fig C), it is noted that the AUC approaches zero with increasing number of features, suggesting a completely unstable hyperplane. This is expected because with number of features much larger than the sample size, the linear SVM problem becomes ill-conditioned, and the margin direction flips unpredictably across folds. The trend is similar to the main study (Fig 7) but amplified with even smaller sample size in the sub-cohort. Nevertheless, before predictive performance deteriorated, the SVM classifier achieved its best interpretable performance with 27 features with ROC-AUC=0.71 and PR-AUC=0.86 (Fig C). By further examining the selected features (Fig D), it is reassuring that statistics of helicity and streamwise vorticity are frequently selected, supporting the findings in the main study and the robustness of the proposed workflow.

Table E. Summary of geometrical features (median [IQR]) with p values.

|  | **Non-vulnerable** | **Vulnerable** | **p value** |
| --- | --- | --- | --- |
| Stenotic Ratio | 0.34 [0.14, 0.37] | 0.30 [0.24, 0.37] | 0.7972 |
| Bifurcation Angle [°], measured at |  |  |  |
| 1mm downstream of bifurcation point | 128 [103, 130] | 102 [97, 106] | 0.3636 |
| 2mm downstream of bifurcation point | 116 [96, 121] | 96 [93, 103] | 0.2398 |
| 3mm downstream of bifurcation point | 110 [89, 113] | 89 [87, 99] | 0.0829 |
| 4mm downstream of bifurcation point | 101 [86, 106] | 83 [82, 88] | 0.1119 |
| 5mm downstream of bifurcation point | 92 [80, 100] | 78 [75, 83] | 0.1119 |
| 7.5mm downstream of bifurcation point | 75 [64, 80] | 66 [59, 72] | 0.2977 |
| 10mm downstream of bifurcation point | 57 [54, 70] | 59 [46, 65] | 0.6064 |
| 20mm downstream of bifurcation point | 31 [29, 42] | 30 [25, 31] | 1 |
| Mean Diameter ICA [mm] | 4.4 [3.6, 5.2] | 4.0 [3.5, 4.9] | 0.6993 |
| Mean Diameter CCA [mm] | 6.3 [5.9, 6.4] | 7.4 [5.7, 7.7] | 0.5185 |
| Min Diameter ICA [mm] | 3.3 [2.3, 3.7] | 3.3 [1.9, 3.9] | 0.8981 |
| Min Diameter ICA/Mean Diameter ICA | 0.79 [0.64, 0.79] | 0.72 [0.64, 0.81] | 1 |
| Mean Diameter ICA/Mean Diameter CCA | 0.57 [0.53, 0.83] | 0.62 [0.51, 0.70] | 0.7972 |
| Tortuosity ROI | 1.1 [1.0, 1.1] | 1.1 [1.0, 1.1] | 0.7972 |
| Step Height Approximation [mm] | 0.77 [0.11, 0.97] | 0.52 [0.37, 1.1] | 0.6064 |
| Reattachment Length [mm] | 20 [1.6, 23] | 13 [7.2, 19] | 0.8981 |
| Distance Bifurcation to ICA Min. Plane [mm] | 2.8 [2.7, 9.4] | 7.9 [2.9, 16] | 0.6993 |
| Distance ICA Min. Plane to Distal Plane [mm] | 4.9 [2.8, 6.2] | 6.1 [4.3, 9.1] | 0.4376 |
| Distance Bifurcation to ROI Last Plane [mm] | 186 [144, 219] | 216 [201, 267] | 0.3636 |

Table F. Summary of hemodynamic features (median [IQR]) at peak systole and diastole with p<0.1 between vulnerable groups.

| **Peak systole** | **Non-vulnerable** | **Vulnerable** | **p value** |
| --- | --- | --- | --- |
| **D Plane** |  |  |  |
| Kurtosis Helicity | -0.76 [-0.78, -0.74] | -1.0 [-1.1, -0.93] | 0.012 |
| **L Plane** |  |  |  |
| Mean Streamwise Vorticity [1/s] | 0.0044 [0.0040, 0.011] | -0.016 [-0.042, 0.0013] | 0.042 |
| Skewness Streamwise Vorticity | -0.12 [-0.62, -0.076] | 0.74 [0.46, 1.5] | 0.0599 |
| Median Streamwise Vorticity [1/s] | 0.012 [0.0083, 0.099] | -0.047 [-0.055, -0.015] | 0.042 |
| **ROI** |  |  |  |
| Skewness Streamwise Vorticity | 1.8 [1.8, 7.8] | 11 [6.6, 13] | 0.042 |
| Median Streamwise Vorticity [1/s] | 0.0078 [0.0054, 0.0098] | -0.00030 [-0.0080, 0.0016] | 0.0829 |
|  |  |  |  |
| **Diastole** | **Non-vulnerable** | **Vulnerable** | **p value** |
| **F Plane** |  |  |  |
| Kurtosis Velocity | -1.1 [-1.2, -1.1] | -0.82 [-1.1, -0.67] | 0.0829 |
| MAD Streamwise Vorticity [1/s] | 0.012 [0.012, 0.028] | 0.033 [0.025, 0.10] | 0.0599 |
| **M Plane** |  |  |  |
| SD Streamwise Vorticity [1/s] | 0.071 [0.030, 0.096] | 0.13 [0.074, 0.48] | 0.0829 |
| MAD Streamwise Vorticity [1/s] | 0.027 [0.023, 0.044] | 0.074 [0.049, 0.26] | 0.012 |
| Median Vorticity [1/s] | 0.11 [0.11, 0.16] | 0.17 [0.13, 0.49] | 0.0829 |
| **D Plane** |  |  |  |
| SD Streamwise Vorticity [1/s] | 0.022 [0.018, 0.059] | 0.082 [0.031, 0.24] | 0.0829 |
| MAD Streamwise Vorticity [1/s] | 0.015 [0.010, 0.026] | 0.057 [0.019, 0.086] | 0.042 |
| Kurtosis Helicity | -0.20 [-0.58, 1.2] | -0.98 [-1.1, -0.95] | 0.019 |
| MAD Helicity | 0.22 [0.14, 0.23] | 0.27 [0.26, 0.40] | 0.012 |
| **ROI** |  |  |  |
| MAD Vorticity [1/s] | 0.022 [0.021, 0.023] | 0.062 [0.027, 0.13] | 0.0599 |

Fig C. Dependency studies of AUC over number of features (A), ROC-AUC (B), PR-AUC (C), and confusion matrix (D) for the LR classifier. Corresponding figures for the SVM classifier are given in (E-H). Shaded areas in (A) and (E) give the range of 95%CI.

Fig D. Selected features across all LOOCV folds from the SVM classifier for the sub-cohort (n=14) analysis. The mean and 95% confidence interval (CI) of the feature weights are given. Frequency (n) shows the total count a feature was selected across all LOOCV folds. The red dashed line at feature coefficient=0 denotes no effect. Peak systole: sys. Diastole: dia.

Fig E. Correlation coefficient matrix for selected features from the union of LR and SVM classifiers. The maximum off-diagonal correlation among selected features is |r| = 0.74, and the majority of pairs fall well below |r| = 0.5, confirming that no problematic multicollinearity persists among the features retained by the classifiers.
